# From the nest to the world: helicopter parenting and challenges in young adult social integration

**DOI:** 10.3389/fpsyg.2025.1432859

**Published:** 2025-06-05

**Authors:** Yelda Yilmaz, Taner Artan, Farida Gurbanova, Nargiz Aliyeva

**Affiliations:** ^1^Faculty of Health Sciences, Department of Social Work, Istanbul Sabahattin Zaim University, Istanbul, Türkiye; ^2^Faculty of Health Sciences, Department of Social Work, Istanbul University-Cerrahpaşa, Istanbul, Türkiye; ^3^UNEC Social Work and Social Innovations Research Center, Baku, Azerbaijan; ^4^Department of Economics on Social Field, Azerbaijan State University of Economics, Baku, Azerbaijan

**Keywords:** family, fear of intimacy, helicopter parenting, self-determination, social work

## Abstract

**Introduction:**

Helicopter parenting is a new parenting style that has become widespread globally. This study aimed to evaluate the effects of helicopter parenting experiences on Turkish young adults, focusing on self-determination and fear of intimacy.

**Methods:**

A cross-sectional design was used with 800 Turkish young adults. Data were collected using the Personal Information Form, Perceived Helicopter Parenting Attitude Scale, Self-Determination Scale, and Fear of Intimacy Scale.

**Results:**

Results showed a significant negative correlation between perceived helicopter parenting and self-determination. There was also a significant positive correlation between perceived helicopter parenting and fear of intimacy. Furthermore, self-determination mediated the relationship between both maternal and paternal helicopter parenting attitudes and fear of intimacy.

**Discussion:**

The findings suggest the importance of increasing awareness about helicopter parenting and its impact on young adults' future lives.

## Introduction

Parenting style is how parents convey attitudes or authority to their children, creating an emotional context for their behaviors (Clauser et al., 2021). One of them is helicopter parenting. Helicopter parenting was first introduced in 1969 by Dr. Haim Ginott, author of “Between Parent and Teenager” (Ginott, 1969). He identified with a child's description of his mother as a “helicopter,” and this meaning has been carried to the present day (Nelson, 2019). The idea of helicopter parenting, which has become increasingly visible due to changing sociocultural circumstances, represents parents who can make decisions and act on behalf of their children (Gomes and Deuling, 2019). With the influence of the current generation, parents may force their children into competition. It may be related to future anxiety, success, and life expectancy (LeMoyne and Buchanan, 2011).

It is seen that there is not a single concept that meets the meaning of helicopter parenting. Cline and Fay (1990) described “helicopter parent theory” as parents who constantly oversee and intervene to save their children whenever they face diffuculties. This parenting style generally attributed to mothers rather than fathers, carries the sub-message that the world is a “dangerous place” in communication with children and may cause them to feel inadequate (Clarke et al., 2013).

There are some characteristic features attributed to helicopter parents. Helicopter parents can establish authority over their children to feel that their children are safe (Vigdal and Brønnick, 2022). This type of parenting can be described as being highly prepared to provide help, support and solve their children's problems at any time (Segrin and Flora, 2019).

Helicopter parents can be concerned about their children encountering malicious people, given that they believe show there are potential dangers in the world (Srivastav and Mathur, 2020). The care and attention these parents show may prevent their children from establishing relationships and impact their self-esteem (Reed et al., 2016). In this context, helicopter parenting behaviors may prevent their children from making decisions that will shape their lives (Luebbe et al., 2018).

Parental anxiety, fear of danger, childhood trauma, and an environment-related sense of insecurity are contributors to the emergence of helicopter parenting (Glass and Tabatsky, 2014). Such parents' anxiety levels may triggered by factors such as the likelihood of something happening to their children (Gui and Koropeckyj-Cox, 2016).

Children raised by helicopter parents with these attitudes may deal with problems in the transition to adulthood (Bradley-Geist and Olson-Buchanan, 2014). Helicopter parenting can impact their children's fear and anxiety of being criticized and evaluated due to controlling behaviors (Carr et al., 2021). In a study, perceived high parental control triggered their child's fear of negative evaluation (Pakdaman and Mortazavi Nasiri, 2014). At the same time, helicopter parenting is associated with “social withdrawal” in children raised by helicopter parents, which means unwillingness to participate in social environments (Jiao et al., 2024).

Helicopter parenting can have an impact on self-efficacy and self-determination. In the literature examining the relationship between helicopter parenting and self-determination, having helicopter parents has negative effects on decision-making processes (Schiffrin et al., 2019). For this reason, the study aimed to see the ongoing psychological and social effects of helicopter parenting in young adults. It addresses the following research question: “*How does perceived helicopter parenting impact young adults' self-determination and fear of intimacy feelings?”*

### Self-determination

Self-determination refers to individuals', groups', and communities' ability to make decisions independently (Van den Broeck et al., 2016). The theory of self-determination explains that this ability is built around internal and external motivations (Gillison et al., 2019). Intrinsic motivation is explained by the individual's satisfaction and interest in their work. In contrast, external motivation encompasses interacting with the environment and being accepted by those around them (Locke and Schattke, 2019).

The main components of the theory of self-determination, introduced by Deci and Ryan in 1985, are autonomy, competence, and relationship building (Chiu, 2022). Autonomy refers to the ability to make decisions on one's initiative, whereas competence refers to the belief that one can succeed and be sufficient (Sheldon and Deci, 2000). Relationship building is explained by individuals feeling they belong to a group and can connect (Joo et al., 2018). These three components can reinforce and encourage individuals' self-determination behavior (Hsu et al., 2019).

Self-determination is the ability to make choices based on fundamental psychological requirements for growth, having the freedom to do so, and being conscious of one's power and limits (Donald et al., 2020). The hypothesis states that environmental factors impact an individual's propensity for autonomous decision-making. It is associated with the concept of “self awareness,” which refers to the ability of individuals to determine the goals they need for their self development and to be aware of their current strengths and limitations (Eurich, 2018). It prioritizes the “right of choice”, which expresses the feeling that one has the right. The right of choice is the capacity to make decisions based on one's values and preferences free, from external factors (Mikhailova, 2021).

According to the self-determination, parents can increase their children's motivation and well-being (Legate et al., 2019). In this respect, it is seen that family relations and participation can have supportive or destructive effect on children's self-determination (Roth et al., 2019). Although they encourage their kids' competition and entrepreneurial spirit, helicopter parents may create risk-averse people instead of risk-takers (Munawar, 2022). The literature about the relationship between helicopter parenting and self-determination has stated a negative correlation. The findings indicate that this parenting style leads individuals to a sense of inadequacy while reducing self-confidence and sense of autonomy (Kouros et al., 2017). Studies indicate that young adults who perceive their parents as helicopter parents have lower psychological needs such as autonomy, competence, and relatedness (Schiffrin et al., 2021). This is because helicopter parents can effect on their children's autonomy, sense of competence, and decision-making processes (Schiffrin et al., 2019). Similar studies indicated that helicopter parenting can lead to a lack of autonomy and a decreased sense of competence (Sharma and Narula, 2024). Within the scope of the literature, the following hypothesis is proposed:

**H1:** *Perceived helicopter parenting has negative effect on self-determination in young adults*.

### Fear of intimacy

According to Mary Ainsworth's attachment theory, intimacy is a factor in the bond established with parents from infancy, which is strengthened by physical contact and provides pleasure (Bergen, 2022). For Bowlby, the need to build and maintain bonds is a fundamental instinct underpinning attachment (Bowlby, 2012). Fear of intimacy means that people avoid communicating with the people around them and stay away from others for some subconscious reason (Manbeck et al., 2020). In the literature, fear of intimacy is associated with individuals' tendency to hide their feelings, thoughts, actions and shy behavior in social environments (Larson et al., 2015).

An analysis of the relevant literature reveals that people who are capable of managing have certain distinct personality traits, skills, and attitudes (Ginevra et al., 2015). It also shows that self-awareness, internal control, problem-solving abilities, and freedom of choice can raise autonomous self-management. These attitudes and behaviors are not considered independent of childhood experiences. While it is known that parents go through the stages of acquaintance, ownership, and attachment with their babies, it is known that especially mothers establish a close bond with their children and are taught how to conduct mutual relations correctly in this way (Kim et al., 2021).

With parental attitudes, the child can learn to socialize, communicate, and develop empathy for understanding people (Weisskirch, 2018). Research has shown that fear of intimacy is mainly driven by the schema shaped by a parent-child relationship (Chen et al., 2020). When children perceive the parenting style as unhealthy, they will have difficulties developing intimate relationships with others (Bavolek, 2021). It may create fear of intimacy in their future lives. Helicopter parents are not always emotionally available for a child or not responsive to their needs; the child can tend to see themselves as worthless of love and care. They may develop a fear of intimacy (Elhami et al., 2019). In a study that included this view, higher levels of helicopter parenting in emerging adults are associated with stronger beliefs in the advantages of being single and an expected delay in marriage (Willoughby et al., 2015).

Reflections of perceived parenting styles continue, especially during the transition to adulthood (Kuckertz et al., 2021). In particular, the studies examining individuals' fear of intimacy often refer to the parent-child attachment and the care relationship between them (Phillips et al., 2013). It is known that people who are afraid of intimacy withdraw from social environments, feel social anxiety, and are isolated in environments where they socialize and communicate with other people (Barzeva et al., 2019). Academic studies reveal the relationship between avoidance behavior and fear of intimacy with loneliness in this context (Maitland, 2020).

Due to its psychosocial effects on individuals, helicopter parenting and fear of intimacy need to be addresses together. There is no study in the literature that directly addressing helicopter parenting and fear of intimacy. However, the concepts of parenting styles, caring relationships, and parental acceptance-rejection continue to be discussed with fear of intimacy. The hypotheses established by the relevant literature are as follows:

**H2:** *There is a positive correlation between perceived helicopter parenting and fear of intimacy in young adults*.

The relationship between self-determination and fear of intimacy is complex and involves various psychological factors. Self-determination, often linked with self-esteem and self-differentiation, plays a significant role in how individuals perceive and engage in intimate relationships. Self-determination theory promotes openness, confidence and support in relationships (Knee et al., 2013). Studies support the idea that self-determination can increase the quality of established close relationships (Brunell and Webster, 2013). According to the literature, the low level of self-determination seen in individuals causes them to feel less secure, which can also prevent them from establishing closeness (Erol and Orth, 2014).

While fear of intimacy can create an internal conflict, as the desire for connection clashes with the anxiety of vulnerability, self-determination emphasizes autonomy (Han and Lee, 2022). The relationship between fear of intimacy and self-determination is rooted in the tension between autonomy and vulnerability. However, fear of intimacy can disrupt this balance by causing individuals to avoid emotional closeness, perceiving it as a threat to their independence or self-identity (Obeid et al., 2019). Prior research focused on the effect of fear of intimacy that is associated with self-silencing, emotional suppression, and social withdrawal in close relationships (Scigala et al., 2021). At the same time, several studies have documented that young adults who perceive their relationship with their parent as helicopter parent have higher levels of social anxiety and fear of intimacy (Jiao et al., 2024). In relation to the literature presented above, the following mediation hypotheses are proposed:

**H3:** *Young adults' self-determination has a mediation role in the correlation between maternal helicopter parenting attitude and fear of intimacy*.
**H4:** *Young adults' self-determination has a mediation role in the correlation between paternal helicopter parenting attitude and fear of intimacy*.

**Figure 1:**
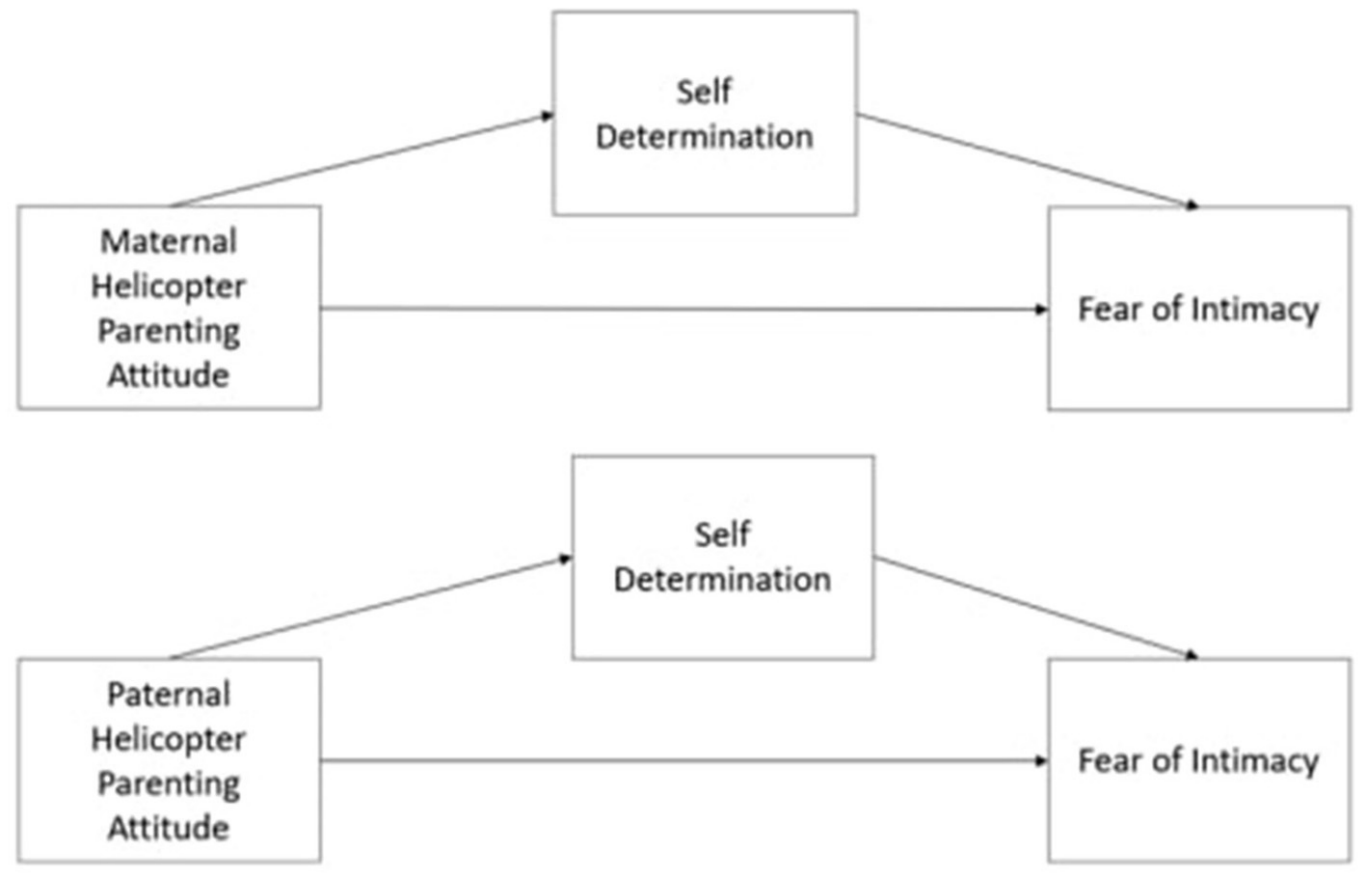
Conceptual diagram of model.

As shown in Figure 1, self-determination is assumed to play a mediating role in the relationship between perceived helicopter parental attitude and fear of intimacy.

## Methodology

### Participants and procedure

The participants of this study consisted of individuals aged 18–45 who remember their childhood experiences with their parents. This age distrubition derives from Daniel Levinson's adulthood development theory (Levinson, 1986). The articles about early adulthood spanned from approximately the end of adolescence until the beginning of middle adulthood. Thus, the period of young adulthood stretches from 18 to 40 or 45 (Arnett et al., 2014).

Young adults who lost their parents during childhood or could not recall those periods were excluded from the study, as the Perceived Helicopter Parenting Scale research rely on childhood memories. The collected data from individuals through an online survey (Google Forms). The online survey was shared on social media channels. No personal information about the participants was included in the questionnaires because they were anonymously completed. It took approximately 10 min to answer the whole question form.

During the data collection phase, the participants were informed about the purpose and parameters of the study, the methodology, and the researchers using the informed permission form linked to the questionnaire. The study initially reached 816 participants. In order to study with participants without outliers, initially IQR method examined was used (Barbato et al., 2011). The IQR method was used to remove outliers (*n* = 16) from the data set using Q1- 1.5 ^*^ IQR as the lower limit and Q3 + 1.5^*^ IQR as the upper limit. Observations outside these boundaries were identified as outliers and removed from the dataset (*n* = 16). The analysis was conducted with 800 participants. The convenience sampling method was used to reach the determined sample number. The approach selected was chosen because it offers an effective way of reaching the participants in the established criteria (Emerson, 2015).

### Measures

In order to collect data in the research, Personal Information Form, Perceived Helicopter Parental Attitude Scale, Self-determination Scale, and the sub-dimension of the Fear of Intimacy Scale “Past-Period Fear of Intimacy” were used. The permission to use the scales was obtained by contacting the researchers who developed and adapted them into Turkish through their institutional e-mails.

#### Personal information form

There are nine questions about age, gender, marital status, education level, income level, family type, number of people in their family, birth order, and pregnancy status.

#### Perceived helicopter parental attitude scale

Perceived Helicopter Parental Attitude Scale is a measuring tool prepared by Yilmaz in 2019 with the Turkish culture in mind and used on four groups of participants between the ages of 13 and 45 (Yilmaz, 2019). The scale consists of four sub-dimensions and 21 likert scale items. The scale sub-dimension about helicopter parenting in “Ethical and Moral Issues” (six items), “Academic/School Life Issues” (five items), “Fundamental Confidence and Life Skills” (six items), and “Emotional-Personal Life” (four items). The scale consists of a 5-point Likert rating system to evaluate helicopter parenting attitudes developed for both mothers and fathers (1 = Never behaves like this, 5 = Always behaves like this). The scale is designed to measure individuals' perceptions of their parents' parenting styles based on their childhood memories. An increasing score for each sub-scale means higher helicopter parenting experiences. As a result of the factor analysis, KMO coefficient (0.89) and for the whole scale's Cronbach's Alpha value was calculated (α = 0.83) and reported (*p* < 0.000). For convergent validity, a correlation between 0.75 and 0.93 was observed between the relevant dimensions of both the Parental Relationship Attitude Scale (Çelik and Öziş, 2016) and the Parental Attitude Scale (Lamborn et al., 1991) and adapted to Turkish by Yilmaz (2000) with both the Mother and Father Forms of the Perceived Helicopter Parenting Scale.

### Self-determination scale

Self-Determination Scale is a measuring tool developed by Ryan and Deci in 1996. The Turkish validity and reliability study of the scale made by Ersoy Kart and Güldü in 2008 consists of nine items and is shaped by two factors (Ryan and Deci, 2020; Ersoy Kart and Güldü, 2008). The scale sub-dimensions were named as “Self Awareness (five items) and “Right of Choice” (four items). The scale was formed in five-point Likert type. Unlike other scales, how the scale is prepared is expressed by pairs of two conditional statements. Accordingly, an item consists of two opposite conditions A and B, and the participants are asked to tick the item they feel close to (Statement A is completely true, statement B is completely true). Option three is appropriate for individuals close to items A and B. As a result of the factor analysis in the study, KMO coefficient (0.72) and for the whole scale's Cronbach's Alpha value was calculated (α = 0.70) and reported (*p* < 0.000). By scrutinizing the factor loadings and methodological notes, we determined that items 1, 4, 6, and 8 required reverse coding. Following this adjustment, we observed a notable improvement in the scale's psychometric properties. Specifically, the Cronbach's Alpha (α) increased to 0.85, reflecting strong internal consistency. Additionally, the correlation between the two subdimensions became both statistically significant and theoretically coherent (*r* = 0.737, *p* < 0.01), thereby reinforcing the appropriateness of the revised scoring procedure.

### Fear of intimacy scale

Fear of Intimacy Scale is a measuring tool developed by Descutner and Thelen in 1991 (Descutner and Thelen, 1991). The scale made by Seyma Elibol and Emine Sevinç Sevi Tok in 2018 consists of 35 items and three factors (Elibol and Sevi Tok, 2018). The scale has three factors: “Imagined Fear of Intimacy” (15 items), “Imagined Openness” (15 items) and “Fear of Past Intimacy” (five items). In this study, we used the 5-item *Fear of Past Intimacy* subscale from the full *Fear of Intimacy Scale* due to its focus on past intimacy experiences, which aligns more closely with the objectives of our research. Similar to how the Perceived Helicopter Parenting Scale assesses individuals' parenting attitudes based on childhood experiences, the *Fear of Past Intimacy* subscale specifically evaluates how past intimacy experiences influence current relational dynamics, particularly fears and anxieties surrounding intimacy. The scale is based on a five-point Likert scale (1 = Not suitable for me at all, 5 = Definitely suitable for me). A high score represents a high level of fear of intimacy. As a result of the factor analysis, KMO coefficient (0.72) and for the whole scale's Cronbach's Alpha value was calculated (α = 0.81) and reported (*p* < 0.000).

### Statistical analysis

A total of 800 forms were used in the study. After cleaning the extreme data in the SPSS, descriptive information and reliability coefficient tables were obtained. The data's normality or non-normality was considered when evaluating the data. According to Tabachnick and Fidell (2013), the results of the normality test results showed that the items had a normal distribution with skewness and kurtosis values between **–**1.50 and +1.50. The study's quantitative data was explained by descriptive analysis, average score calculation, correlation analysis and direct regressions through IBM SPSS 26 program and PROCESS Macro plug-in. Mediation analyses are conducted to go beyond the interaction between variables to understand how other variables affect this interaction. The mediating role of self-determination in the interaction between paternal and maternal helicopter parenting attitudes was analyzed. Model four was presented by Hayes in the SPSS Process Macro plug-in. Model four is the appropriate model to identify the role of the mediating variable in the interaction between the independent and the dependent variables (Hayes, 2022).

## Findings and hypotheses tests

### Descriptive statistics

The sociodemographic information of the participants was presented in the study. It was reported that 72.8% of the participants in the study in Table 1 were female, 27.3% were male, and the average age was 26.34 ± 6.17. Due to the country's inflation rate changes over time and their high levels, participants' subjective assesment of their income status was categorized into five levels: low, below average, average, above average, and high. About half of the participants (48%) had middle income status. This rate is followed by participants who declare low income status (21.1%). A significant portion lived in the nuclear family type (77.1%). While 17% have a large family, 13% have a single-parent family. When the number of people in the family they were raised in was examined, it was found that most of the participants were between five and nine (53.3%). A similar proportion of participants (42.2%) grew up in a family with 1–4 people. Almost all (93%) were placed between the first and fourth children in the family. At the same time, it was concluded that nearly all participants (84.1%) were born due to a desired pregnancy. Only 15.2% of them declared that they were born as a result of an undesired pregnancy.

**Table 1:** Perceptions of young adults in the study as helicopter parents.

**Variable**	***n***	**%**
Number of mothers perceived as helicopter mothers	575	71.9
Number of mothers not perceived as helicopter mothers	225	28.1
**Total (** ***N*** **)**	800	100
Number of fathers perceived as helicopter fathers	339	42.4
Number of fathers not perceived as helicopter fathers	461	57.6
**Total (** ***N*** **)**	800	100

n = Number of participants.

Table 1 presents findings on whether young adults in the study perceive their mothers as helicopter parents. The “Perceived Helicopter Parenting Scale” cut-off values were used to assess whether the individuals had helicopter parents. Accordingly, participants scoring 56 points and above on the scale thought they had a helicopter parent. Most respondents (71.9%) perceive their mother as a helicopter parent. Only 28.1% of respondents believe they do not have helicopter parents. Looking at the findings about whether the young adults in the study perceived their father as a helicopter parent, it is seen that 42.4% perceived their father as a helicopter parent. In comparison, 57.6% did not perceive their father as a helicopter parent.

### Means, standard deviations, and correlation analyses

Table 2 shows the correlation, mean, and standard deviation values of the main variables and sub-dimensions. There is a positive correlation between fear of intimacy and maternal helicopter parenting attitude (*r* = 0.147, *p* < 0.01), and between fear of intimacy and paternal helicopter parenting attitude (*r* = 0.107, *p* < 0.01). In contrast, there is a negative correlation between paternal helicopter parenting attitude and self-determination (*r* = −0.134, *p* < 0.01). The relationship between fear of intimacy and self-determination is negative (*r* = −0.374, *p* < 0.01). It should be noted that some correlations presented in this table are statistically significant but many are relatively small in size.

**Table 2:** Means, standard deviations and correlations.

**Variables**	**1**	**2**	**3**	**4**	**5**	**6**	**7**	**8**	**9**	**10**	**11**	**12**	**13**	**14**
1. MHPA	1													
*2. EAMI*	0.812^**^	1												
*3. BCALS*	0.838^**^	0.629^**^	1											
*4. AASLI*	0.807^**^	0.572^**^	0.568^**^	1										
*5. EAPL*	0.766^**^	0.482^**^	0.504^**^	0.577^**^	1									
6. PHPA	0.547^**^	0.433^**^	0.472^**^	0.411^**^	0.417^**^	1								
*7. EAMI*	0.455^**^	0.548^**^	0.389^**^	0.289^**^	0.257^**^	0.802^**^	1							
*8. BCALS*	0.468^**^	0.335^**^	0.560^**^	0.276^**^	0.299^**^	0.839^**^	0.618^**^	1						
*9. AASLI*	0.410^**^	0.314^**^	0.311^**^	0.451^**^	0.274^**^	0.855^**^	0.648^**^	0.619^**^	1					
*10. EAPL*	0.413^**^	0.245^**^	0.263^**^	0.314^**^	0.518^**^	0.788^**^	0.494^**^	0.523^**^	0.618^**^	1				
11. SD	−0.167^**^	−0,262^**^	−0.191^**^	−0.075^*^	0.016	−0.134^**^	−0.265^**^	−0.157^**^	−0.082^**^	0.028	1			
*12. ROC*	−0.154^**^	−0.245^**^	−0.170^**^	−0.060	0.016	−0.145^**^	−0.265^**^	−0.159^**^	−0.095^**^	0.011	0.909^**^	1		
*13. SA*	−0.152^**^	−0.235^**^	−0.181^**^	−0.072^*^	0.012	−0.110^**^	−0.239^**^	−0,137^**^	−0.058	0.034	0.938^**^	0.737^**^	1	
14. FOI	0.147^**^	0.187^**^	0.135^**^	0.057	0.079^*^	0.107^**^	0.172^**^	0.120^**^	0.027	0.026	−0.374^**^	−0,363^*^	−0.332^**^	1
Mean	44.65	8.31	10.39	8.93	8.65	37.78	7.30	8.61	7.93	6.94	27.24	11.00	13.82	2.60
SD	11.04	2.53	3.04	2.44	2.74	10.23	2.31	2.71	2.49	2.46	7.51	3.50	3.97	0.99

^**^p <0.01, ^*^p <0.05.

MHPA, Maternal helicopter parenting attitude; EAMI, Ethical and moral issues; BCALS, Basic confidence and life skills; AASLI, Academic and school issues; EAPL, Emotional and personal life; PHPA, Parental helicopter parenting attitude; SD, Self-determination; ROC, Right of choice; SA, Self awareness; FOI, Fear of intimacy.

### Direct effects

The effect of the dependent variables on the study's independent variable is given in Table 3. In the table, three different models valid for helicopter mother and father attitudes were created with the appropriate model.

**Table 3:** Main regression effects of helicopter parenting attitudes on fear of intimacy.

	**Model 1: SD**			**Model 2: FOI**			**Model 3: FOI**		
**Variable**	**B**	**SE**	***p***	**B**	**SE**	***p***	**B**	**SE**	***p***
(Constant)	32.331	1.092	0.000	3.550	0.195	0.000	2.015	0.144	0.000
MHPA	−0.113	0.023	0.000	0.007	0.003	0.008	0.013	0.003	0.000
SD			0.000	−0.047	0.004	0.000			0.000
F		22.977			68.910			17.683	
*p*		<0.001			<0.001			<0.001	
R^2^		0.028			0.147			0.021	
(Constant)	30.971	1.007	0.000	3.713	0.183	0.000	2.215	0.133	0.000
PHPA	−0.098	0.025	0.000	0.005	0.003	0.082	0.010	0.003	0.002
SD				−0.048	0.004	0.000			
F		14.632			66.669			9.207	
*p*		<0.001			<0.001			<0.01	
R^2^		0.018			0.143			0.011	

MHPA, Maternal helicopter parenting attitude; FOI, Fear of intimacy; PHPA, Parental helicopter parenting attitude; SD, Self-determination; F, Analysis of variance (ANOVA); p, Significant value; R^2^, Determination coefficient; B, Unstandardized regression coefficient.

The effect of perceived helicopter mother attitude on the fear of intimacy in Model 1, which was first seen, was analyzed. Accordingly, the effect of the perceived maternal helicopter attitude on self-determination was negative (B = −0.113, *p* < 0.001).

In Model 2, the effects of perceived maternal helicopter attitude and self-determination on fear of intimacy were analyzed. The effect of self-determination on fear of intimacy was found to be negative (B = −0.047, *p* < 0.001), while the effect of maternal helicopter attitude was positive and significant (B = 0.007, *p* = 0.008). In Model 3, the effect of maternal helicopter attitude on fear of intimacy was again significant, indicating a positive total effect (B = 0.013, *p* < 0.001).

Similarly, in Model 1 for paternal helicopter attitude, it was found to have a negative effect on self-determination (B = −0.098, *p* < 0.001). Model 2 examined the effects of perceived paternal helicopter attitude and self-determination on fear of intimacy. The paternal helicopter attitude showed a positive but marginally significant effect (B = 0.005, *p* = 0.082), while self-determination had a significant negative effect (B = −0.048, *p* < 0.001). Finally, Model 3 showed that the total effect of paternal helicopter attitude on fear of intimacy was positive and statistically significant (B = 0.010, *p* < 0.01).

### Indirect effects

The scale has a separate scoreable structure for both mothers and fathers. Thus, analyses were supposed to be appropriate for constructing the analyses as perceived maternal and paternal helicopter parenting attitudes. Direct regression analyses were conducted between the variables to assess the relationships underlying the mediation model, with the results reported in Table 4. Accordingly, the effects of perceived maternal and paternal helicopter parenting attitudes on self-determination, as well as the effect of self-determination on fear of intimacy, were found to be statistically significant.

**Table 4:** Total, direct and indirect effects of helicopter parenting attitude on fear of intimacy.

	**Effect type**		**Unstand**.	**SE**	**LLCI**	**ULCI**
	Total effects of MHPA on FOI		0.0132	0.0031	0.0070	0.0194
	Direct effects of MHPA on FOI		0.0078	0.0030	0.0020	0.0137
**Indirect effects of maternal helicopter parenting attitude on fear of intimacy via self-determination**						
MHPA >	SD >	FOI	0.0054	0.0013	0.0029	0.0081
	Total effects of PHPA on FOI		0.0103	0.0034	0.0037	0.0170
	Direct effects of PHPA on FOI		0.0056	0.0032	−0.0007	0.0119
**Indirect effects of paternal helicopter parenting attitude on fear of intimacy via self-determination**						
PHPA >	SD >	FOI	0.0048	0.0014	0.0021	0.0075

MHPA, Maternal helicopter parenting attitude; FOI, Fear of intimacy; PHPA, Parental helicopter parenting attitude; SD, Self-determination; Unstand., Unstandardized coefficients; SE, Standard error; LLCI, Lower limit confidence interval; ULCI, Upper limit confidence interval.

As presented in Table 4, the mediation analysis revealed that self-determination significantly mediated the relationship between helicopter parenting attitudes and fear of intimacy. Specifically, the indirect effect of maternal helicopter parenting attitudes on fear of intimacy through self-determination was statistically significant (γ = 0.0054, SE = 0.0013, 95% CI [0.0029, 0.0081]). Similarly, the indirect effect of paternal helicopter parenting attitudes on fear of intimacy via self-determination was also significant (γ = 0.0048, SE = 0.0014, 95% CI [0.0021, 0.0075]).

### The results of the proposed research model and hypotheses

**Figure 2:**
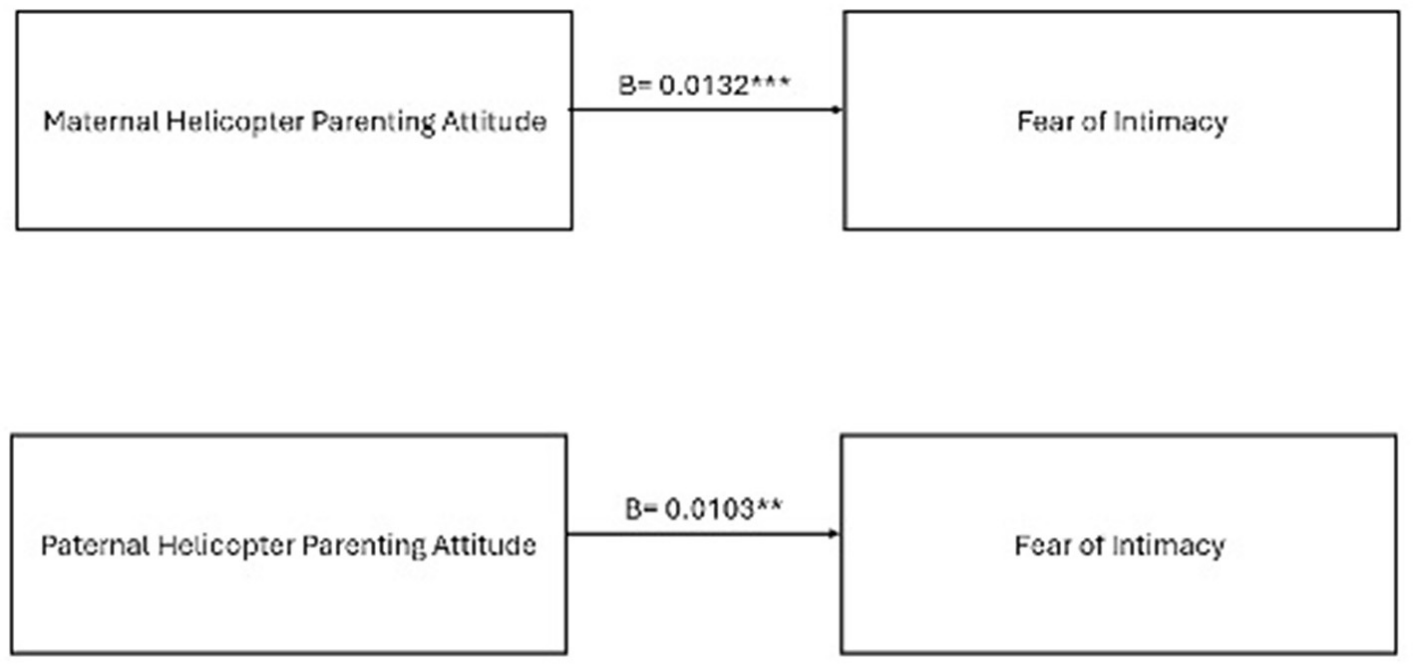
The total effects of the proposed research model. **p < 0.01. ***p < 0.001.

**Figure 3:**
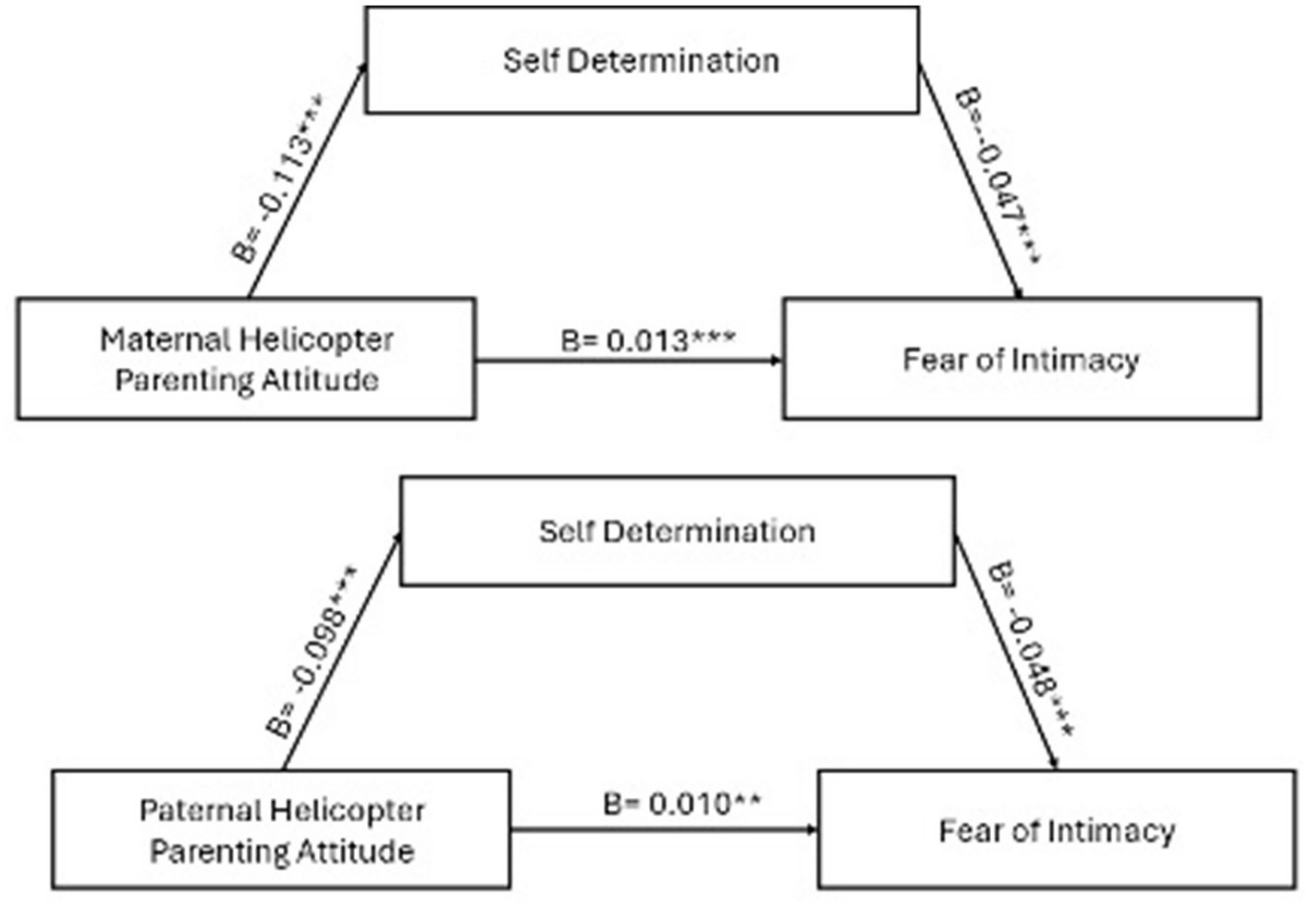
The results of the proposed research model. **p < 0.01. ***p < 0.001.

The direct, indirect, and moderation effects shown in Figure 1 were tested. The coefficients and significance levels are placed in Figures 2, 3. According to the results, the hypotheses between H2 and H4 were fully supported. The hypotheses and research questions were evaluated in detail in the discussion part.

## Discussion

Helicopter parenting has been the phenomenon of different focused studies since its prevalence increased in the 2000s and beyond. However, no study evaluates the relationship between fear of intimacy, self-determination, and having helicopter parents. Therefore, this study will support the current literature by underlining the prevalence of helicopter parenting and seeing its effects in future projections. This research aims to explore the effects of helicopter parenting on young adults, with particular attention to their self-determination and fear of intimacy. As in the current literature, the study's findings suggest that perceived helicopter parenting attitudes can affect young adults. A highlight of the study is that having helicopter parents allows young adults to study their self-determination and fear of intimacy together. We examined whether or not self-determination mediated the relationship of helicopter parenting with fear of intimacy.

### Prevalence of having helicopter parenting

When evaluating the prevalence of helicopter parenting attitudes in the study, it is observed that the prevalence of perceived helicopter mothers is higher than that of fathers by young adults. Contrary to the findings, a study that systematically compiled field papers on the subject concluded that mothers were more accepting and supportive of autonomy than fathers (Yaffe, 2020).

However, some studies have found that mothers play a more dominant role than fathers, which is consistent with the findings of this study, explaining that mothers spend more time with their children regarding gender roles (Axpe et al., 2019). These studies suggest that mothers' helicopter parenting attitudes are perceived more than fathers (Pistella et al., 2020; Rote et al., 2020). Another study evaluating helicopter mothers' and fathers' characteristics noted that helicopter mothers call and ask their children more than fathers (Kömürcü Akik and Alsancak Akbulut, 2023). In a study comparing helicopter parenting attitudes in the United States and China, helicopter mothers are primarily in the USA, unlike China (Hwang et al., 2022). It is also reported in the studies that helicopter mothers are more effective than fathers, especially regarding decisions regarding education and work life (Fingerman et al., 2020).

As can be seen, there is no consensus with a standard sample regarding gender and cultural differences. However, this finding can be related to the fact that mothers may assume a more significant role in childcare than fathers. Generally, primary caregiving mothers assume a greater share of the emotional care responsibility for childcare than primary caregiving fathers (Pinho and Gaunt, 2021). Additionally, the fact that 72.8% of the study's sample is female may have prompted them to recall memories related to their mothers, from whom they are likely to have taken role models in their childhood. Studies show that mothers play a role in developing their daughters' beliefs, attitudes, social norms, and behaviors, including acting as role models (Santarossa and Woodruff, 2020).

Looking at the prevalence of helicopter parenting, the study revealing the weekly hours parents spend raising their children reported that six OECD countries (Spain, Canada, Italy, the UK, Netherlands, and the USA) experience helicopter parenting with increasing momentum (Doepke and Zilibotti, 2019). Studies of a similar nature confirm that helicopter parenting has become increasingly common over time (Ahmed and Mingay, 2023; Doepke, 2021; Howard et al., 2022; Jung et al., 2019; Karunaharan et al., 2021; Miller-Ott, 2016; Moorhouse, 2021; Shaki et al., 2022). It is necessary to infer from the findings that helicopter parenting is an increasingly common trend globally.

### Effects of helicopter parenting on self-determination and fear of intimacy

Regression analyses showed that the perceived helicopter parental attitudes restricted young adults' ability to make decisions independently. In a study of 40 college students, helicopter parenting reduced students' self-efficacy (Kwon et al., 2017). In another study of students, helicopter parents prevented children from taking the initiative and did not allow them to mature (Love et al., 2020).

The academic literature is reported that the effects of helicopter parenting are also seen when their children become adults, and it may cause them to have difficulties in taking responsibility and establishing their own families (Cui et al., 2019). Within the framework of the analyses, helicopter parenting was closely related to fear of intimacy. Helicopter parenting attitudes impacted teen flirting relationships, according to a study of 202 participants looking at the relationship between perceived helicopter parental attitude and rapport (Kim, 2022). In a longitudinal study of 982 people examining the effect of parental control on socialization, it was observed that individuals who thought their parents controlled them tended to hide and stay away from others while socializing (Tilton-Weaver et al., 2010). A meta-analysis of 95 studies on helicopter parents, revealed that they were afraid of potential risks and led a lifestyle focused on their children's behavior (McLaughlin, 2020). In this respect, helicopter parents' attitudes and behaviors may influence children's ability to build social relationships.

At the beginning of the study, we asked a research question: “*How does perceived helicopter parenting impact young adults' self-determination and fear of intimacy feelings?”* We explored whether self-determination mediates the relationship between helicopter parenting and fear of intimacy. It was determined that general helicopter parenting attitudes had a significant positive association with the fear of intimacy and self-determination. However, it is important to note that while helicopter parenting is associated with fear of intimacy, this does not necessarily imply that fear of intimacy leads to loneliness, nor can it represent all social interaction difficulties. Helicopter parenting can contribute to difficulties in forming intimate relationships, but fear of intimacy is just one aspect of broader social challenges and other factors. For instance, personal experiences and coping mechanisms should be considered.

## Limitations

The current study has some limitations. This research was carried out cross-sectionally. Different research results can be obtained at other times because self-determination, perceived helicopter parenting, and fear of intimacy are dynamic processes. It is advised that longitudinal research be carried out in the future. This study's second limitation was that the sample consisted of individuals between 18 and 45. Access to this broad age group required the use of an online questionnaire. This situation highlights the study's inclusion limits. The data was ultimately gathered inside a unique national culture. This might raise some concerns about the results' generalizability. In this regard, the validity of the findings will be increased by doing the study again with individuals who have helicopter parents in various nations. The findings are limited by the homogeneity of the sample, which may not fully capture the diversity of experiences related to helicopter parenting. Expanding the sample to include participants from varied backgrounds would enhance generalizability. The reliance on self-reported measures introduces the potential for social desirability bias. Future research should incorporate multi-informant approaches or objective assessments of parenting behaviors. Given the increasing prevalence of helicopter parenting as a parenting style, it is essential to carry out various research.

## Conclusion and some implications

The findings of this study examine how young adults perceive helicopter parents and their effects on fear of intimacy and self-determination behavior. By examining how perceived helicopter parenting affects young adults' autonomy and their ability to build close relationships, the study contributes to a deeper understanding of this parenting style's psychological and social implications. The findings underscore the importance of fostering independence and self-determination in young adults, emphasizing the potential long-term effects of helicopter parenting. Since helicopter parenting is a significant concept that evaluates the parent-child relationship, it was expected to contribute to the different academic areas. The following is a summary of the findings and the answer to the research question created within the literature on helicopter parenting.

In the study, perceived helicopter parenting reduces the self-determination of young adults. Since the social work profession predicts that people can know their needs best, individuals should be active in decision-making (Timms, 2018). However, helicopter parents can act with their worldviews and expectations.

Perceived helicopter parenting can affect young adults from building intimacy. Based on the knowledge that behavior patterns in the family may contribute to fear of intimacy (Han and Lee, 2022; Hwang et al., 2023). For this reason, helicopter families' social support mechanisms are thought to be transferred to their children in a way (Hwang et al., 2023).

Based on the underlying conclusion of the study, the following recommendations have been proposed for future studies.

Future research can explore the prevalence and impact of helicopter parenting across diverse cultural and socioeconomic contexts. Comparative studies can reveal how cultural norms and values shape helicopter parental behaviors and their effects on young adults.

A longitudinal approach can offer deeper insights into the long-term effects of helicopter parenting, tracking young adults' developmental trajectories over time and identifying critical periods for intervention.

Experimental research is needed to evaluate the effectiveness of interventions designed to mitigate the effects of helicopter parenting. Programs that enhance communication skills, foster independence, and encourage parental self-awareness could be assessed.

With the increasing integration of digital tools into parenting, studies should examine how technology (e.g., constant digital monitoring) amplifies or alters the dynamics of helicopter parenting.

## Data Availability

The datasets presented in this article are not readily available due to participant restrictions. Specifically, participants requested that information related to their families be used solely for the researchers' analysis. Requests to access the datasets can be directed to YY at yelda.yilmaz@izu.edu.tr.
